# The impact of COVID-19 on sexual behaviors of young women and men: A protocol for systematic review and meta analysis

**DOI:** 10.1097/MD.0000000000024415

**Published:** 2021-02-26

**Authors:** Qi Zhang, Hua Lu, Fangyuan Li, Xinyun Li, Tong Wang, Qian Yang, Ling Mi

**Affiliations:** a College of Clinical Medicine, Chengdu University of Traditional Chinese Medicine, NO.37 Shi-er-qiao Road; b Department of Gynecology, Hospital of Chengdu University of Traditional Chinese Medicine, NO.39 Shi-er-qiao Road; c Maternal and Child Reproductive Hospital affiliated to Chengdu University of Traditional Chinese Medicine, NO.17, Section 4, South Renmin Road, Chengdu, Sichuan Province, PR China.

**Keywords:** COVID-19, meta-analysis, sexual behaviors, systematic review

## Abstract

**Background:**

The worldwide impact of COVID-19 has reached all spheres of human health. Individuals may also experience unique changes in their sexual behaviors during the COVID-19 self-isolation/social distancing period. Studies in many countries have assess the effects of the pandemic on sexual behavior, or quality of sexual life. However, no systematic review has comprehensively explored the association between COVID-19 and the sexual behaviors of young women and men to date. This systematic review and meta-analysis protocol aims to ascertain the association between COVID-19 and sexual behaviors of young women and men that may get targeted interventions to improve health and well-being of young people's sexual health.

**Methods:**

This systematic review and meta-analysis will be reported following the PRISMA guidelines. Observational designs (including cross-sectional, case-control, and cohort) will be eligible. Studies eligible for inclusion must contain participants aged 15 to 45 in any country affected by the pandemic of COVID-19. The search will be conducted in the following databases, including PubMed, Cochrane Library, EMBASE, EBSCO, Ovid, WHO COVID-19 database, China National Knowledge Internet (CNKI), WanFang Data, Chinese Scientific and Technological Journal Database (VIP), and Chinese Biomedical Databases (CBM). A pre-designed search strategy of medical subject heading (MeSH) terms and free words for the concepts “COVID-19” and “sexual behaviors” will be used. Two authors will independently complete literature screening, data extraction, and risk of bias assessment. Disagreements will be resolved by consensus with a third reviewer. The reviewer will follow the PECOS steps (population, exposure, comparator, outcomes, and study design) to obtain eligible extraction items. The risk of bias and quality of included studies will be assessed using RevMan 5.3. We will assess heterogeneity according to the *I*^2^ statistics. If there is substantial heterogeneity in the included trials, subgroup analysis will be carried out to seek the potential causes.

**Ethics and dissemination:**

It is not necessary to obtain ethical approval as we will use data from published articles. The findings of this systematic review will be published in a peer- reviewed journal.

**PROSPERO registration number:**

PROSPERO 2020: CRD42020190867.

## 1 Introduction

COVID-19 is a respiratory virus that is transmitted through large respiratory droplets and direct contact with infected secretions.^[1]^ Since December 2019, the novel coronavirus was first discovered, the high transmission potential has contributed to the global coronavirus disease 2019 (COVID-19) pandemic in 2020.^[2]^ As of mid-November, the global cumulative number of confirmed cases of COVID-19 has exceeded 50 million reported to WHO. According to statistics, the number of confirmed cases of COVID-19 globally increased from 30 million to 40 million in 32 days, and from 40 million to 50 million in just 20 days. This series of data shows that the COVID-19 coronavirus has not quieted but accelerated its spread worldwide.^[3]^ Look back the past year, the COVID-19 global pandemic has resulted in disorder to the health-care systems and deterioration of social life. Series of strict control measures including self-isolation or quarantine adopted by the government had caused lots of negative effects.^[4]^ The population experienced restricted activity, panic, poor mental health, loss of relatives, life-threatening conditions, unemployment, reduced income, and separation from family or partners.^[5]^ Thus, 1 behavior that may consequently be impacted by self-isolation/social distancing is that of sexual behavior.^[6]^ In times of pandemic, the focus on sex-related issues is a striking sign of how important sexual services and science are, even when survival is a priority.^[7]^ To provide advice or inform further research on sexual behavior during periods of high stress or anxiety during self-isolation/social alienation, it is important to understand levels and correlates of sexual activity.

On one hand, sexuality is one of the most private things to do with close people which the degree of intimacy is great. On the other hand, sexuality is complex, and the term “sexual behavior” encompasses a large spectrum of actions including orientation, partnerships, attitudes, identity, beliefs, behaviors, and activity.^[8]^ Individuals are believed successful sexual activity is an important activity for promoting physical health and emotional well-being, and usually to use sex as means to cope with dysfunctional mood. Evidence shows that sexual activity can regulate endocrine and immune systems^[9]^ and prolong life.^[10]^ Additionally, successful sexual activity can also help people relax body, release stress, fall asleep, even in depressed and high-anxiety patients.^[11]^ Regular sexual activity has advantages for women, including regulating the menstrual cycle, relieving dysmenorrhea, and reducing the risk of endometriosis.^[12]^ However, long-term sexual dysfunction can be an early sign of some diseases such as diabetes, hypertension, or cardiovascular.^[13,14]^ In daily life, it may constitute some interpersonal conflict due to deteriorating self-esteem or partner relationships.^[15]^ Therefore, sexual behavior as a basic aspect of health is of current interest to clinicians and sexologists.

At the height of the COVID-19 epidemic, it has be confirmed the impact of the COVID-19 on sexual behavior.^[16]^ A probable correlation is that the Angiotensin-Converting Enzyme-2 (ACE2) receptors as an entry point used by COVID-19 to invade the respiratory system is a constitutive product of adult Leydig cells which can cause involvement of the testicles and reduction of testosterone secretion.^[17]^ In China, the researchers found that both sexual activities and sexual satisfaction of young men and women declined, and low sexual desire and unsatisfying partner relationships were significant factors affecting sexual activities.^[18]^ According a study from NBC News, among more than 9000 people, 47% said COVID-19 infection had affected their sex lives negatively.^[19]^ Likewise, Two scholars in Istanbul, Turkey published that during the COVID-19 pandemic, sexual desire, and frequency of intercourse significantly increased in female, whereas quality of their sexual life sharply decreased.^[20]^ This is consistent with previous research that pressure can impact the sexual desire and frequency of sexual intercourse.^[21]^ Under this background, scholars in many countries have given clinical recommendations for safe sexual activity during the COVID-19 coronavirus pandemic.^[22]^

However, there is no systematic review and meta-analysis of studies reporting on the relationship between COVID-19 and sexual behaviors of young women and men. So our reviewer teams begin to do this job and aim to ascertain the association between COVID-19 and sexual behaviors of young women and men that may get targeted interventions to improve health and well-being of young people's sexual health.

## 2 Methods and analysis

### 2.1 Study registration

This review protocol is registered on the International Prospective Register of Systematic Reviews (PROSPERO) with the registration number: CRD42020190867. The systematic review and meta-analysis was designed in accordance with the Preferred Reporting Items for Systematic Reviews and Meta-Analyses Protocols (PRISMA-P) statement^[23]^ and the Meta-analyses of Observational Studies in Epidemiology (MOOSE) Checklist was followed.^[24]^

### 2.2 Database search and search strategy

The search will be conducted in the following databases, including PubMed, Cochrane Library, EMBASE, EBSCO, Ovid, WHO COVID-19 database, China National Knowledge Internet (CNKI), WanFang Data, Chinese Scientific and Technological Journal Database (VIP), and Chinese Biomedical Databases (CBM). The data and information will be retrieved by 2 independent reviewers (QZ and FYL), and differences will be resolved by discussion with a third reviewer (QY). The databases will be searched from November 2019 to April 2021.

Each database will be searched by using medical subject heading (MeSH) terms and free words (in the title and abstract) for the concepts “COVID-19” and “sexual behaviors” (combined with the Boolean logic operation “AND”). The detailed search strategies and search terms used for PubMed are shown in Table 1. The corresponding adjusted search strategy and search term will be applied to other databases. In order to improve the deficiency of the electronic database, we will continue to search the clinical trial registration platforms (i.e., Clinicaltrials.gov) and gray literature at the same time. If necessary, we will contact the first author or corresponding author by E-mail or telephone to complete the data.

**Table 1:** Search strategy for PubMed.

Number	Search terms of query
#1	“COVID-19” [Supplementary Concept,Mesh]
#2	“2019 novel coronavirus disease”[Title/Abstract] OR “COVID19”[Title/Abstract] OR “covid 19 pandemic”[Title/Abstract] OR “sars cov 2 infection”[Title/Abstract] OR “covid 19 virus disease”[Title/Abstract] OR “2019 novel coronavirus infection”[Title/Abstract] OR “2019 ncov infection”[Title/Abstract] OR “coronavirus disease 2019”[Title/Abstract] OR “coronavirus disease 19”[Title/Abstract] OR “2019 ncov disease”[Title/Abstract] OR “covid 19 virus infection”[Title/Abstract]
#3	“Sexual Behavior”[MeSH Terms]
#4	“behavior sexual”[Title/Abstract] OR “sexual activities”[Title/Abstract] OR “sexual activity”[Title/Abstract] OR “activities sexual”[Title/Abstract] OR “activity sexual”[Title/Abstract] OR “sex behavior”[Title/Abstract] OR “behavior sex”[Title/Abstract] OR “oral sex”[Title/Abstract] OR “sex oral”[Title/Abstract] OR “sexual orientation”[Title/Abstract] OR “orientation sexual”[Title/Abstract] OR “sex orientation”[Title/Abstract] OR “premarital sex behavior”[Title/Abstract] OR “behavior premarital sex”[Title/Abstract] OR “anal sex”[Title/Abstract] OR “sex anal”[Title/Abstract]
#5	(#1 OR #2) AND (#3 OR #4)

### 2.3 Inclusion and exclusion criteria

#### 2.3.1 Study design

We will include the studies with observational designs (including cross-sectional, case-control, and cohort) concerning the association between COVID-19 and sexual behaviors of young women and men, which meet all inclusion criteria. Report on the studies in English or Chinese is eligible to be included only.

#### 2.3.2 Participants

Young women and men aged 15 to 45 in any country affected by the pandemic of COVID-19 will be included. Pregnant and lactating women, COVID-19 positivity, sexual partner with COVID-19 infection, homosexual or bisexual individuals, mental illness including depression or personality disorders, and using the medicine that reduces libido for the previous 3 months will be excluded.

#### 2.3.3 Exposure

The pandemic of COVID-19.

#### 2.3.4 Comparators

Sexual behaviors of young women and men prior to the pandemic of COVID-19.

#### 2.3.5 Outcome measures

Outcomes were obtained using Female Sexual Function Index (FSFI) questionnaires, Female Sexual Distress Scale (FSDS) questionnaires, or self-made questionnaires to assess the changes in people's sexual behavior. Self-made questionnaires should explicitly reported at least 1 of the following: sexual frequency, sexual desire, risky sexual behavior, sexual satisfaction, sexual partners, frequency of masturbation, frequency of pornography use.

### 2.4 Literature screening

**Figure 1:**
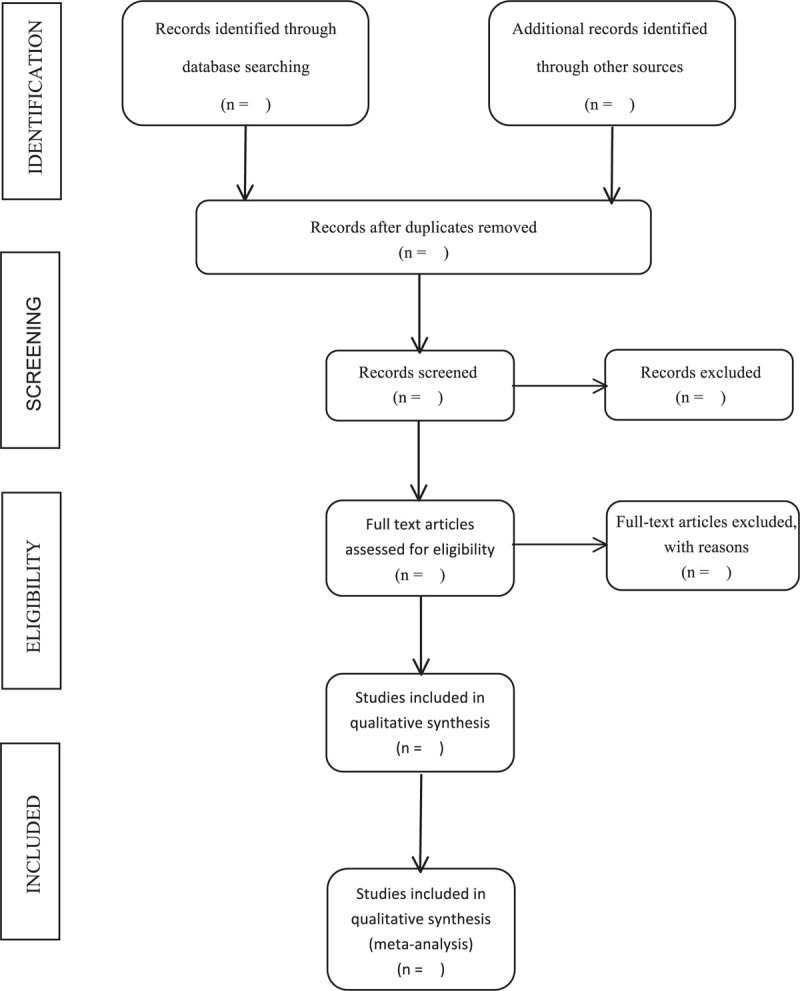
The PRISMA literature screening flow chart. PRISMA = Preferred Reporting Items for Systematic Reviews and Meta-Analyses.

All the articles from the database searches will be managed by the software Endnote X9 (Boston, USA). Firstly, by comparing the title, author, year, journal name, volume, page number and other information, the duplicates will be removed. Two skillful reviewers (QZ and FYL) will independently screen titles and abstracts in parallel to move bibliography that clearly does not meet the inclusion criteria into the exclusion folder. Records that meet the requirements of the preliminary review will then be taken forward to full-text screening. The reviewers will scrutinize the content of the study design in the full text of the potentially eligible full-text reports one by one to decide whether to include or not, and record the reasons for the exclusion in detail in Excel. The process of screening will be repeatedly cross-check, if there is any uncertain problems, it should be discussed with the third reviewer (QY) to resolve. The entire process of literature screening is summarized as a flowchart in Figure 1.

### 2.5 Data extraction

Before formal literature extraction, we will make a standardized literature extraction table according to the decided literature data extraction content, and select 5 to 6 pieces of literature to verify the table. Formal literature extraction will be performed by 2 authors independently (QZ and FYL). Any differences will be resolved through discussion or the participation of a third reviewer (QY). We will follow the PECOS steps (Population, Exposure, Comparator, Outcomes, and Study design) to obtain eligible extraction items. The data items will be extracted is as follows:

**Population:** Participants’demographic characteristics (e.g., gender, age, partner’ age, country, ethnic, marriage status, education level, current financial situation, accommodation, partner relationship), inclusion and exclusion criteria, comorbidities (e.g., Vaginal infections), alcohol or drug intake.

**Exposure:** Pandemic of COVID-19, number of included subjects, details of COVID-19 severity (e.g., Isolated state, COVID-19 infection status of participants and those around them).

**Comparators:** Sexual behaviors (e.g., sexual frequency, sexual desire, risky sexual behavior, sexual satisfaction, sexual partners) before pandemic.

**Outcomes:** Identification of sexual behaviors outcomes (e.g., FSFI, FSDS, or self-made questionnaires), COVID-19-related impact on sexual health (e.g., sexual frequency, sexual desire, risky sexual behavior, sexual satisfaction, sexual partners, frequency of masturbation, frequency of pornography use), information gathering time, survey platforms (e.g., Questionnaire Star).

**Study characteristics:** Literature title, literature number, full name of a journal, year of publication, country/region, first author, author email, objective, study design, sample size.

### 2.6 Risk of bias assessment

Refer to the Newcastle- Ottawa Scale for observational studies to evaluate the quality of the literature included in this study.^[25,26]^ Two well-trained reviewers (QZ and TW) will be blinded to the titles, journals, authors of the studies to assess the risk of bias of each included study and jointly check the evaluation results. The bias will be divided into 3 levels (high risk, low risk, or unclear) for individual elements from 8 domains. A high score will be considered as a low risk of bias, (i.e., the NOS score ≥7 indicates high-quality). Inconsistent evaluation results will be settled by consensus. Any disagreements that cannot be resolved will be discussed with a third party (FYL). After that, we will use RevMan 5.3 to pool the data for analysis and generate the bias assessment graphs.

### 2.7 Statistical analysis

#### 2.7.1 Assessment of heterogeneity

The ability to perform a meta-analysis and the decision on which model to use will depend on the evaluation of the heterogeneity, which will be realized according to the Cochrane Handbook criteria through the *I*^2^ statistic. We consider that an *I*^2^ value less than 50% demonstrates a non-substantial level of heterogeneity, and a fixed-effect model^[27]^ will be adopted for the analysis; >50% values represent considerable heterogeneity, and a random-effects model^[28]^ will be used.

#### 2.7.2 Data synthesis and analysis

If the heterogeneity between the included studies is within the acceptable range, we will consider performing a meta-analysis. If the included studies are not eligible for meta-analysis due to significant heterogeneity, other characteristics and results will be summarized narratively to synthesize. We will summarize the evidence for the impact of COVID-19 on sexual behaviors of young women and men.

#### 2.7.3 Subgroup and sensitivity analyses

If there is substantial heterogeneity in the included trials, subgroup analysis will be carried out to seek the potential causes. If the data retrieved is sufficient, we will conduct a subgroup analysis according to subject characteristics (e.g., age, country, ethnicity, marital status, education level, current financial situation). If significant heterogeneity still existed after subgroup analysis, a sensitivity analysis was performed to assess the robustness of summary estimates by excluding the study one by one.

### 2.8 Ethics and dissemination

Ethical approval is not required as all the data used in this systematic review will be extracted from published literature. The results will be disseminated by publication of the manuscript in a peer-reviewed journal and for national and international presentations.

## 3 Discussion

The outbreak of COVID-19 worldwide poses a huge threat to human health including sexual health.^[29]^

Satisfactory sexuality benefits males and females physically and emotionally and having a favorable impact on their quality of life.^[30]^ Previous reports have shown that mass disasters can affect people's sexual behavior. For example, women's sexual intercourse frequency, sexual satisfaction and desire for children both decreased following the Wenchuan earthquake.^[31]^ Although many people attempt to research the impact of COVID-19 in all areas of sexuality,^[7]^ no review has comprehensively explored the association between COVID-19 and the sexual behaviors of young women and men to date. The protocol will facilitate an understanding of the impact of COVID-19 on young people’ sexual behaviors. We hope an increased understanding of the relationship between COVID-19 and the sexual behaviors of young women and men may be able to deliver better practices for times of crisis, and above all, to ensure human sexual rights despite adversity. Since no limited by race or country, the study will have broad representativeness. We will conduct the systematic review and meta-analysis through 2 independent reviewers, and consult with a third researcher when no consensus is reached or data collection is inconsistent, thus reducing the potential limitations inherent in the systematic review and meta-analysis. In addition, we will follow existing guidelines, PRISMA-P statement, the MOOSE Checklist, and recommendations from the Cochrane Collaboration Handbook.

## Author contributions

**Conceptualization:** Qi Zhang, Hua Lu, Fangyuan Li.

**Data curation:** Qi Zhang, Tong Wang.

**Formal analysis:** Qi Zhang, Fangyuan Li, Qian Yang.

**Funding acquisition:** Hua Lu, Ling Mi.

**Investigation:** Fangyuan Li, Tong Wang.

**Methodology:** Xinyun Li.

**Project administration:** Qi Zhang, Hua Lu, Fangyuan Li.

**Resources:** Qi Zhang, Fangyuan Li, Qian Yang.

**Software:** Xinyun Li, Ling Mi.

**Visualization:** Ling Mi.

**Writing – original draft:** Qi Zhang.

**Writing – review & editing:** Hua Lu.
